# Molecular Detection and Prevalence of Equine Piroplasmosis and Other Blood Parasites in Equids of Western Aegean Türkiye

**DOI:** 10.3390/vetsci12090826

**Published:** 2025-08-27

**Authors:** Selin Hacilarlioglu, Huseyin Bilgin Bilgic, Tulin Karagenc, Heycan Berk Aydin, Hasan Toker, Hakan Kanlioglu, Metin Pekagirbas, Serkan Bakirci

**Affiliations:** 1 Department of Parasitology, Faculty of Veterinary Medicine, Aydın Adnan Menderes University, 09017 Aydın, Türkiye; selin.uner@adu.edu.tr (S.H.); hbilgic@adu.edu.tr (H.B.B.); tkaragenc@adu.edu.tr (T.K.); heycanberkaydin@gmail.com (H.B.A.); tokerisg@gmail.com (H.T.); metin.pekagirbas@adu.edu.tr (M.P.); 2 Medical Laboratory Techniques Program, Department of Medical Services and Techniques, Aydın Vocational School of Health Services, Aydın Adnan Menderes University, 09010 Aydın, Türkiye; hkanlioglu@adu.edu.tr

**Keywords:** equine blood samples, equine piroplasmosis, *Anaplasma phagocytophilum*, *Trypanosoma* spp., *Leishmania* spp., Molecular detection, PCR, Türkiye

## Abstract

Equine piroplasmosis (EP), caused by *Theileria equi* and *Babesia caballi*, is a tick-borne disease posing significant threats to equine health and the horse industry worldwide. Other vector-borne blood parasites, including *Anaplasma phagocytophilum*, *Trypanosoma* spp., and *Leishmania* spp., can also infect horses; however, their prevalence remains poorly characterized in Türkiye. This study aimed to determine the molecular prevalence of *T. equi*, *B. caballi*, *A. phagocytophilum*, *Trypanosoma* spp., and *Leishmania* spp. in equids from the Western Aegean Region of Türkiye. Blood samples were collected from 388 clinically healthy equines across İzmir, Aydın, Denizli, and Muğla provinces. Species-specific PCR assays were performed, and *T. equi* and *B. caballi* were detected in 24.74% (96/388) and 12.89% (50/388) of samples, respectively, with co-infections in 3.09%. *T. equi* and *B. caballi* infections were detected in horses from all four sampled provinces—Aydın, İzmir, Denizli, and Muğla—except for *B. caballi*, which was not found in any samples from Muğla. No samples tested positive for *A. phagocytophilum*, *Trypanosoma* spp., or *Leishmania* spp. Prevalence significantly varied by province, breed, age, and sex (*p* < 0.05). This study demonstrates the considerable prevalence of *T. equi* and *B. caballi* in Western Türkiye, underlining the need for routine screening and vector control programs. The absence of other parasites suggests limited circulation; however, continued surveillance remains crucial to safeguard equine health and prevent disease spread.

## Simple Summary

In this study, blood samples from 388 equines in Western Aegean Türkiye were analyzed for the presence of *Theileria equi*, *Babesia caballi*, *Anaplasma phagocytophilum*, *Trypanosoma* spp., and *Leishmania* spp. using molecular methods. *T. equi* was detected in 24.74% (96/388) of the samples, and *B. caballi* in 12.89% (50/388). Infections with *T. equi* and *B. caballi* were detected across all four provinces—Aydın, İzmir, Denizli, and Muğla—with the exception of *B. caballi*, which was not detected in any of the samples from Muğla. None of the other blood parasites investigated were detected in any of the samples. These results indicate that *T. equi* is widely distributed in the region and that carrier animals may play an important role in the transmission and spread of this disease.

## 1. Introduction

Equines, including horses, mules, donkeys, and zebras, are distributed worldwide, and their economic value varies significantly between countries [1]. Throughout history, Equidae have been an important component of animal husbandry in Türkiye, where they have been raised primarily for agricultural, transportation, military, and sporting purposes [2]. Despite ongoing use in some regions, Türkiye’s equine population declined sharply, from 495,543 in the 1990s to 70,360 in 2024, largely due to mechanization in agriculture and transport [3]. However, the sector remains economically important, particularly in the breeding and management of sport horses. Equines are susceptible to a range of diseases, many of which are vector-borne and show nonspecific signs (e.g., fever, anemia, edema). These overlapping signs complicate diagnosis as they are shared by several vector-borne diseases—including *Anaplasma phagocytophilum*, *Trypanosoma* spp. (Surra and Dourine), *Borrelia burgdorferi* (Lyme disease), *Leishmania* spp., West Nile virus, and the causative agents of equine piroplasmosis (EP)—*Babesia caballi* and *Theileria equi* [2,4,5,6,7]. These diseases continue to pose health and economic challenges, particularly in regions where competent arthropod vectors are endemic.

EP is one of the most important vector-borne diseases of equids. It is caused primarily by *T. equi*, *T. haneyi*, and *B. caballi*, which are intra-erythrocytic parasites belonging to the phylum Apicomplexa [8]. Transmission occurs through ixodid ticks such as *Amblyomma*, *Hyalomma*, *Haemaphysalis*, *Dermacentor*, *Ixodes*, and *Rhipicephalus* [8,9,10]. The geographical distribution of these tick vectors overlaps with the areas where the disease is endemic, notably in countries within the Afrotropical, Neotropical, and Palearctic regions [11]. Acute disease can be fatal if left untreated. Chronically infected animals serve as long-term reservoirs, facilitating parasite transmission. Notably, *T. equi* can persist lifelong in untreated hosts. Therefore, the identification and monitoring of chronically infected equids are crucial to prevent further transmission to naïve animals [12]. Although *B. caballi* can also establish chronic infections, horses can clear the parasite within approximately four years in the absence of reinfection [12,13,14]. Preventing the introduction of EP into non-endemic areas remains a priority to safeguard equine health and international movement.

Equine granulocytic anaplasmosis (EGA), caused by *Anaplasma phagocytophilum*, is a zoonotic tick-borne disease of equids [15]. *A. phagocytophilum* primarily affects horses during the colder months, with prevalence peaking from late autumn to early spring [16]. Depending on the geographic region, various tick species act as vectors for *A. phagocytophilum*, including *Ixodes*, *Dermacentor*, *Rhipicephalus*, *Hyalomma*, and *Haemaphysalis* species [16]. EGA is characterized by fever, reluctance to move, lethargy, ataxia, distal limb edema, lameness, and hematological abnormalities such as thrombocytopenia, anemia, lymphopenia, and neutropenia in horses. It generally resolves spontaneously within 7–14 days [17,18].

Equines have also been increasingly recognized as incidental or potential reservoir hosts for *Leishmania* spp., especially in regions endemic for canine leishmaniasis. Equine cutaneous leishmaniasis (ECL) has been sporadically reported in various parts of the world. The first documented case occurred in a horse from Argentina, followed by reports in donkeys and mules from Brazil and Venezuela [19,20,21]. To date, phlebotomine sand fly species, including *Phlebotomus* spp. and *Lutzomyia* spp., have been reported to feed on domestic animals, including equines, and to transmit *Leishmania* parasites to horse populations without exhibiting species discrimination [21]. In some endemic regions, infection rates in donkeys have even surpassed those in humans and domestic dogs, suggesting a possible epidemiological role for equids [21]. In Europe, *L. infantum* has been identified in equids from Spain, Portugal, and Italy, particularly in areas with high canine infection rates [22,23,24,25,26]. Additionally, *L.* (*Mundinia*) *martiniquensis*, formerly known as *L. siamensis*, has been detected in horses in the United States, Switzerland, and Germany—countries where canine leishmaniasis is not endemic [7,27]. Given the increasing number of reported cases and the close proximity of equids to humans and dogs, particularly in rural and peri-urban settings, investigating *Leishmania* infections in these animals is essential to better understand their potential role as sentinels, incidental hosts, or secondary reservoirs.

*Trypanosoma* spp., particularly *Try. evansi* and *Try. vivax*, are among the most pathogenic and economically significant species affecting horses, with a broad distribution across Africa, the Middle East, Asia, and Latin America [6,28,29]. *Try. evansi*, the causative agent of Surra, is mechanically transmitted, mainly via blood-feeding insects such as horseflies (Tabanidae) and stable flies (*Stomoxys*) [30,31]. Surra, a zoonotic vector-borne disease, is characterized by clinical signs including fluctuating fever, weakness, lethargy, anemia, severe weight loss, petechial hemorrhages on the eyelids and vulvar mucosa, abortion, movement disorders, and edema [28,29,32,33,34].

In Türkiye, most haemoparasitological studies have focused on *T. equi* and *B. caballi*, with reported seroprevalence rates ranging from 0–34.6% and 12.8–56.8%, respectively. Molecular studies report 2–38.8% for *B. caballi* and 3–50% for *T. equi* [35]. *A. phagocytophilum* has been investigated in only three studies—one serological [36] and two molecular [35,37]. However, there is a notable lack of data on other vector-borne haemoparasites such as *Trypanosoma* and *Leishmania* spp. Given the potential zoonotic importance and epidemiological implications of these pathogens, it is essential to expand surveillance efforts beyond *Theileria* and *Babesia*. In light of this knowledge gap, the present study aims to perform a molecular screening for *T. equi*, *B. caballi*, *A. phagocytophilum*, *Trypanosoma* spp., and *Leishmania* spp. in equids from the Western Aegean Region of Türkiye.

## 2. Materials and Methods

### 2.1. Sampling and Study Area

**Figure 1:**
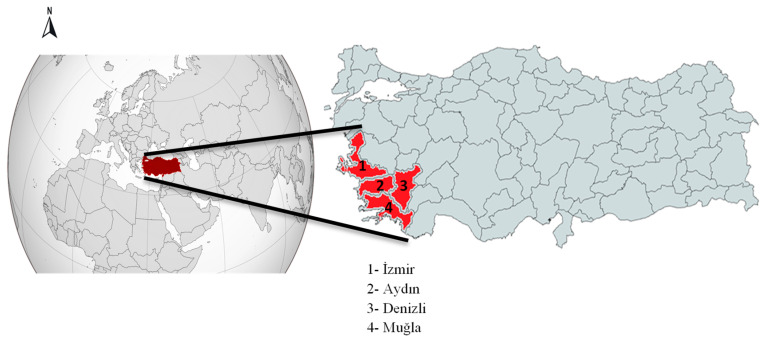
Geographic distribution of equine samples collected from the Western Aegean Region of Türkiye.

This study was conducted on clinically healthy horses, donkeys and mules residing in the Western Aegean Region of Türkiye. To ensure that the study sample accurately represented the population, we estimated the sample size based on the number of animals in each sampling region (Table S1). The sample size was calculated using OpenEpi software (Version 3.01, Emory University, Atlanta, GA, USA) [38]. A total of 388 samples were collected from the provinces of İzmir (*n* = 84), Aydın (*n* = 177), Denizli (*n* = 53), and Muğla (*n* = 74). Figure 1 shows the geographical location of the study area within Türkiye. For statistical analysis, age, sex, breed of each animal, and sampling location were considered. At sampling, none of the animals showed clinical disease signs, and no ticks were observed. Additionally, no information was available regarding their clinical history or any previous antiparasitic treatments. Blood samples were collected from the jugular vein into sterile vacuum tubes containing EDTA (BD Vacutainer^®^, Franklin Lakes, NJ, USA) from at least the minimum calculated number of animals. Genomic DNA was extracted from blood samples using the Wizard Genomic DNA Purification Kit (Promega, Madison, WI, USA), following the manufacturer’s instructions. Extracted DNA was resuspended in 100 μL elution buffer and stored at −20 °C until analysis. Control DNA samples used in this study were sequence-confirmed reference isolates, labeled according to species and origin, including *T. equi*/Ankara, *B. caballi*/Ankara, and, *L. infantum*/Aydın from Türkiye, *A. phagocytophilum*/United Kingdom, and *Try. evansi*/Tunisia.

### 2.2. Detection of T. equi, B. caballi, A. phagocytophilum, Trypanosoma *spp*., and Leishmania *spp*.

All 388 samples were screened with an array of species-specific PCRs for the presence of *T. equi*, *B. caballi*, *A. phagocytophilum*, *Trypanosoma* spp., and *Leishmania* spp. Details of primer pairs for each species are given in Table 1. PCR reactions were performed in a final volume of 50 μL containing 10 mM Tris–HCl (pH 8.3), 50 mM KCl, 2 mM MgCl_2_, 0.001% gelatin, 250 μM of each deoxynucleotide triphosphate, 0.25 U of VitaTaq DNA polymerase (Procomcure Biotech, Bergheim, Austria), 0.5 µM of forward and reverse primer, and 1 µL of DNA template. For the second round of nested PCR (nPCR) targeting *B. caballi* and *A. phagocytophilum*, 1 µL of template DNA (first round PCR product) was used. The reactions were performed using an automatic thermal cycler (Techne, TC-512, Staffordshire, UK), and reaction conditions are summarized in Table 1. For each reaction, 10 μL of PCR product was electrophoresed on a 1.5% agarose gel containing 10 μL/mL SybrGreen (SafeView™, ABM Inc., Richmond, BC, Canada) in Tris-acetate-EDTA (TAE) buffer at 100 V. The sizes of the amplified products were estimated using a 100 base pair DNA ladder (GeneDireX^®^, GeneDireX Inc., Taichung, Taiwan), and the products were visualized on a UVP EC3 Bio-Imaging System with VisionWorksLS software (Version 6.8, Analytik Jena US, Upland, CA, USA).

### 2.3. Sequencing of Amplified PCR Products

In order to confirm the specificity and accuracy of the PCR assay, Sanger sequencing was performed on three randomly selected positive samples for each parasite species, and these were submitted to a commercial sequencing service (Atlas Biotechnology Laboratory, Ankara, Türkiye). These included three samples obtained from *T. equi*-positive animals, targeting the 18S rRNA gene with an expected product size of 435 base pairs using the BEC-UF2 and EQUI-R primers (Table 1), and three samples from *B. caballi*-positive animals, targeting the *rap-1* gene with an expected product size of 222 base pairs, using the BcaN-F and BcaN-R primer pairs (Table 1). The resulting sequences were analyzed using BLASTn (www.ncbi.nlm.nih.gov, accessed on 21 May 2025), version 2.16.0 and compared with reference sequences in the GenBank database to confirm their identity.

### 2.4. Statistical Analysis

Statistical analyses were performed using SPSS version 22.0 (SPSS Inc., Chicago, IL, USA). Data were first assessed for normality, and one-way analysis of variance (ANOVA) was used for normally distributed variables. For non-normally distributed data, the Kruskal–Wallis test was applied. Post hoc tests were conducted to determine which specific groups differed when a statistically significant result was obtained. Results are expressed as mean ± standard error (SE), and a *p* value of <0.05 was considered statistically significant.

### 2.5. Ethics Statement

The study was approved by the institutional animal Ethics Committee of the Aydın Adnan Menderes University (Protocol number: 64583101/2018/067) and conducted according to national guidelines conforming to European Directive 2010/63/EU.

## 3. Results

### 3.1. Prevalence of T. equi, B. caballi, A. phagocytophilum, Trypanosoma *spp*. and Leishmania *spp*.

In the present study, a total of 388 samples were collected from equines, of which 215 (55.41%) were classified as adults (>3 years), 42 (10.83%) as young stock (≤3 years), and 131 (33.76%) as unknown age. The sample population comprised 135 males (34.79%), 88 females (22.68%), and 165 (42.53%) with unknown sex, thus providing a comprehensive representation of the species (Table 2). Some of the recorded data, including the age, sex, and breed of the animals, could not be located for analysis due to an unexpected circumstance. As a result, these data points were treated as missing values to avoid inaccuracies in the statistical analysis. All samples were screened by PCR for the presence of *T. equi*, *B. caballi*, *Anaplasma* spp., *Trypanosoma* spp., and *Leishmania* spp. infections. However, only *T. equi* and *B. caballi* were detected; all samples tested negative for *A. phagocytophilum*, *Trypanosoma* spp., and *Leishmania* spp. PCR revealed 134 animals (34.54%) positive for at least one parasite, including 12 (3.09%) with co-infections. *T. equi* was the most prevalent parasite, identified in 96 horses (24.74%), whereas *B. caballi* was detected in 50 horses (12.89%). There was no statistically significant difference in prevalence between *T. equi* and *B. caballi* (*p* = 0.896) among sampled animals.

**Figure 2:**
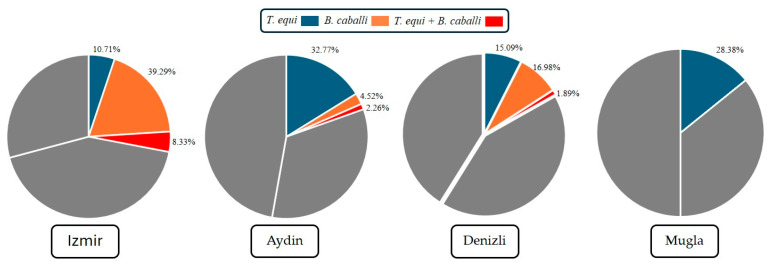
Distribution of *Theileria equi* and *Babesia caballi* samples among provinces.

Geographically, *T. equi* and *B. caballi* infections were identified in horses from all four sampled provinces—Aydın, İzmir, Denizli, and Muğla—with the exception of *B. caballi*, which was not detected in any of the samples from Muğla. Figure 2 compares the molecular prevalence of *T. equi* and *B. caballi* among the four provinces sampled in the Western Aegean Region of Türkiye. The distribution of *T. equi* across the study area showed considerable variation; the highest infection rate was observed in Aydın (58/177, 32.77%), followed by Muğla (21/74, 28.38%), Denizli (8/53, 15.09%) and İzmir (9/84, 10.71%). Similarly, the prevalence of *B. caballi* also varied by region, ranging from null in Muğla to 39.29% in İzmir. The highest frequency of *B. caballi* infection was recorded in İzmir (33/84, 39.29%), followed by Denizli (9/53, 16.98%) and Aydın (8/177, 4.52%). The differences in prevalences of *T. equi* (*p* < 0.001) and *B. caballi* (*p* < 0.001) among provinces were statistically significant (Table 3), indicating that geographic location may play a role in the epidemiology of EP in the Western Aegean Region of Türkiye (Figure 2).

*Theileria equi* prevalence significantly differed among breeds (*p* < 0.001) (Table 2). However, no significant differences were observed between age groups (≤3 years vs. >3 years; *p* = 0.871) or sex (females: 24/88, 27.27% vs. males: 35/135, 25.93%; *p* = 0.645). In contrast, the distribution of *B. caballi* infections showed notable differences based on age, sex, and breed. The infection was more prevalent (*p* = 0.004) in females (8/88, 9.09%) compared to males (7.41%). Furthermore, when comparing different age groups, the positivity rate was higher in individuals older than three years (19/215, 8.83%) than in those aged three years or younger (3/42, 7.14%) (*p* = 0.002) (Table 2). In the present study, significant differences were observed in the prevalence of *T. equi* and *B. caballi* infections among different horse breeds and species (*p* < 0.001). *T. equi* was detected across most horse breeds and donkeys, with the highest infection rates recorded in local horses (12/20, 60.0%), ponies (2/4, 50.0%), donkeys (9/21, 42.86%), and Ambler horses (27/76, 35.52%). In contrast, lower prevalence rates were noted in Arabian (11.43%) and English (2.86%) horses. Notably, *T. equi* was not detected in Haflingers. Regarding *B. caballi*, infections were primarily found in local (4/20, 20.0%) and Arabian (12/70, 17.14%) breeds, while no cases were detected in Ambler horses, donkeys, mules, or Haflingers. Although the sample size for some breeds was limited, these findings suggest that breed may influence susceptibility or exposure to EP pathogens, potentially due to differences in vector contact, geographical distribution, or husbandry conditions.

### 3.2. Sequence Results

To confirm the accuracy of the PCR assay, six representative positive samples (three *T. equi* and three *B. caballi*) were randomly selected, and subjected to sequence analysis. The BLAST analysis of the three sequences obtained from *T. equi*-positive samples revealed 99–100% identity with sequences previously reported from Türkiye (MG569900), Sudan (AB515311), and Israel (KX227639). Sequences from the three *B. caballi*-positive samples showed 99% identity to reference sequences from Brazil (MG906584) and Sudan (LC514709).

## 4. Discussion

The significance of EP is underscored by the global mobility of horses, particularly those with substantial economic value that are transported for participation in international equestrian sporting events [44,45]. Many countries enforce strict import regulations to prevent the introduction of positive animals into their territories. These countries have established unified and widely accepted policies at both federal and state levels for the identification and management of seropositive horses [46,47,48]. In Türkiye, horse importation is regulated through licensing systems requiring certification of freedom from specific diseases such as equine infectious anemia, African horse sickness, dourine, glanders, anthrax, and equine viral arteritis [49]. However, an important tick-borne disease; EP, caused by *T. equi* and *B. caballi* and transmitted by ticks belonging to the genera *Dermacentor*, *Rhipicephalus*, and *Hyalomma* [50], is not included in the list of infections evaluated during the importation process. EP is widespread globally and the disease most commonly occurs in its chronic form, which is characterized by nonspecific clinical signs such as lethargy, partial anorexia, weight loss, and poor performance. Consequently, infected animals frequently become asymptomatic carriers, sustaining transmission [9,10,50].

Traditionally, diagnosis of EP has relied on microscopic examination of Giemsa-stained blood smears [51], but this method lacks sensitivity, especially in chronic infections with low levels of parasitemia [9,50]. Serological techniques offer improved sensitivity for detecting subclinical infections, but they cannot differentiate between past and current infections [52]. In contrast, PCR-based molecular techniques allow for highly sensitive and specific detection of active infections and enable species-level identification [39,53]. The prevalence of *T. equi* varies significantly across different regions and continents, affected by diagnostic methods as well as local epidemiological factors such as vector density, host behavior, and strategies for tick control [51]. Globally, molecular prevalence rates range from 0.8–96.8% for *T. equi* and from undetectable to 78.0% for *B. caballi* [54,55].

In Türkiye, several studies have reported a wide range of prevalence levels, from 0% to 85%, using molecular techniques [35,56,57,58]. In the present study, conducted on 388 equids from the Western Anatolia Region of Türkiye, the molecular prevalence of *T. equi* and *B. caballi* was found to be 24.74% and 12.89%, respectively. Mixed infections were detected in 3.09% of the animals. Consistent with most previous findings, *T. equi* was found to be more prevalent than *B. caballi* in this study, which can be attributed to the lifelong carrier status of *T. equi* infections [51]. While interpreting the molecular prevalence results for *T. equi*, it is important to consider potential diagnostic limitations of the assay used in this study. Although the assay used in this study targets the 18S rRNA gene region specific to *T. equi*, previous studies have shown high sequence similarity between *T. equi* and *T. haneyi* [59,60]. It should be noted that cross-amplification cannot be completely excluded, and some of the *T. equi*–positive results in this study may potentially represent *T. haneyi*. Due to the limited sequencing data and the lack of *T. haneyi*–specific PCR assays, we were unable to confirm or exclude this species. Considering that *T. haneyi* is increasingly reported in various regions and may be misidentified as *T. equi* in 18S-based assays, future studies in this region should include *T. haneyi*–specific molecular testing [59,61]. Additionally, sequencing a larger proportion of positive samples would help clarify the occurrence and prevalence of this species. Given the widespread presence of competent tick vectors in Türkiye [62], the detection and elimination of persistent *T. equi* (and potentially *T. haneyi*) infections are critical steps toward preventing the re-emergence and spread of the disease. The role of chronically infected equids as reservoirs for tick transmission, as proposed by Scoles and Ueti (2015) [10], underscores the need for routine molecular screening, especially in regions with active equine movement and trade. Integrating molecular diagnostics with tick surveillance and vector control programs would strengthen current prevention strategies and help mitigate the risk of new outbreaks.

In the present study, due to an inexplicable circumstance, some of the recorded data, including age, sex, and breed of the animals, could not be retrieved for the analysis. As a result, these data points were treated as missing values to prevent any inaccuracies in the statistical analysis. Thus, the following statistical conclusions are only valid for animals with complete data. In this study, *T. equi* was detected in all the provinces examined—Aydın, İzmir, Denizli, and Muğla—while *B. caballi* was not detected in the Muğla. The prevalence of both parasites varied significantly among sampled provinces (*p* < 0.001). The observed differences may be influenced by breed distributions and horse usage (e.g., heavy labor), which affect tick exposure, especially in Muğla where tick control is less rigorously applied. Earlier studies identified a positive correlation between age and *T. equi* or *B. caballi* prevalence, indicating higher risk in older animals [63,64]. In this study, while no statistically significant difference in the rate of *T. equi* infection was observed between age groups, *B. caballi* infections were significantly more common in animals over 3 years of age (*p* = 0.002). Although this finding may reflect the larger sample size in this age group, it contrasts with previous reports indicating higher *B. caballi* prevalence in younger animals [8]. In general, *T. equi* prevalence tends to increase with age, due to its ability to establish lifelong carrier states. In contrast, *B. caballi* infections may be cleared over time as host immunity develops [51]. Similarly, *T. equi* prevalence did not vary by sex, whereas *B. caballi* was significantly more common in females (*p* = 0.004). The influence of host sex on susceptibility to protozoan infections, including *T. equi* and *B. caballi*, has been debated. Some studies have linked sex differences in infection rates to the modulatory effects of sex hormones on immune responses [65]. For example, in *Leishmania* infections, androgens and estrogens modulate the Th1/Th2 balance, affecting host susceptibility [66]. However, data on EP are inconsistent. While several studies report higher infection rates of *T. equi* in females [11,63,67,68], others have found no significant association between sex and seropositivity for either *T. equi* or *B. caballi* [12,58,69].

Breed has been identified as a potential risk factor affecting seroprevalence of EP [67]. Both parasites have also been detected in other equids, including donkeys and mules [8,70,71,72,73]. In this study, the prevalence of both parasites differed significantly among breeds (*p* < 0.001). Local donkeys exhibited the highest prevalence of *T. equi* (9/21, 42.86%) after local horse breeds (12/20, 60%). Conversely, *B. caballi* was not detected in donkeys. This finding suggests that the more persistent *T. equi* infections follow a similar course in donkeys, which may act as carriers of EP, potentially affecting their health and work performance [74]. Although donkeys are considered more resistant than horses [74], this assumption remains insufficiently supported, as data on domestic equines—particularly donkeys and mules—are less comprehensive than those available for horses. The lower infection rates observed particularly in sport horses may reflect reduced exposure to tick infestation as well as more effective management and parasite control programs. In other words, the higher prevalence observed in mules and donkeys has been attributed to their frequent outdoor activities, particularly their involvement in daily wood transportation from forests [64]. This may also be associated with their prolonged exposure to pasture environments, which increases the likelihood of tick bites [51].

Equine granulocytic anaplasmosis (EGA), caused by *A. phagocytophilum*, is a tick-borne disease with a worldwide distribution [15,16]. In Türkiye, *A. phagocytophilum* infections have been reported at varying prevalence rates not only in horses but also in cattle [75,76,77], sheep [78,79,80], goats [81], and dogs [82]. However, data on its prevalence in horses remain limited. Previous studies reported a seroprevalence of 8.6% [37], while molecular prevalence rates were 6.6% [35] and 6.4% [37]. In contrast to these findings, *A. phagocytophilum* was not detected in the present study. This variation in findings may be attributed to differences in geographical distribution, sample size, seasonal factors, or tick infestation rates in the study areas.

Among salivarian trypanosomes, *Try. evansi* and *Try. vivax* are responsible for Surra and Nagana diseases, respectively. They cause fatal infections in a wide range of domestic and wild animals, including camelids, horses, cattle, buffalo, small ruminants, pigs, carnivores, deer, gazelles, and elephants [29,30,32,83]. Although, *Try. evansi* has been reported in Türkiye [29], no subsequent studies have examined the presence of the parasite in equines so far. In the present study, no positive samples were detected for *Try. evansi* or *Try. vivax*. These findings align with the absence of reported clinical trypanosomiasis cases in horses from Western Anatolia. This suggests that *Try. evansi* is likely not present in this area. The lack of molecular detection of *Try. evansi* and *Try. vivax* may be attributed to parasite clearance, seasonal variations in parasitemia, or parasitemia levels below the detection threshold of the PCR assay used [32]. Nevertheless, considering that outbreaks of *Try. evansi* have been reported in neighboring countries [84,85], located approximately 1500 km away from the sampling sites in this study, strict control measures regarding the importation and movement of animals from these regions remain crucial, especially due to the widespread distribution of blood-sucking insect vectors capable of transmitting these parasites.

*Leishmaniasis* is a zoonotic disease caused by species of the genus *Leishmania*, which are obligate intracellular protozoa transmitted by blood-sucking female sand flies [86]. The disease affects not only humans but also a variety of domestic and wild animals [7]. Recent studies have reported *Leishmania* infections in equines in both endemic [7,20] and non-endemic regions, and sporadic cases of equine cutaneous leishmaniasis caused by *L. infantum* [22,87] or *L. martiniquensis* [27]. Visceral and cutaneous leishmaniasis caused by *L. infantum* and *L. tropica* are endemically seen in the Aegean region of Türkiye [88]. Although several studies in European countries have investigated the role of different farm animals, including horses, in the transmission of leishmaniasis, no such studies have been conducted in Türkiye to date. In the present study, PCR assays targeting the LT1 gene region of *Leishmania* were performed on blood samples collected from equines in the sampling area; however, no *Leishmania* DNA was detected in any of the samples. PCR, as a molecular diagnostic technique, has higher sensitivity when tissue samples such as bone marrow, lymph nodes, spleen, or culture isolates are used, compared to blood samples [89]. Thus, reliance on blood samples may partly explain the absence of positive detections. It is evident that both sample type and sample size are critical factors that should be considered in studies aiming to determine the prevalence of leishmaniasis in equids, particularly in endemic areas. Indeed, researchers have reported that in endemic regions, the prevalence of infection in donkeys may exceed that observed in humans and domestic dogs [21].

In the present study, blood samples were collected from the jugular vein of clinically healthy animals for the molecular detection of all targeted parasites. However, it should be noted that the choice of sampling site can significantly influence the detection sensitivity of blood parasites. Previous studies have recommended jugular venipuncture for the detection of *T. equi*, *B. caballi*, and *A. phagocytophilum*, particularly in healthy animals [12,14,90]. In contrast, for *Try. evansi*, sampling from peripheral capillary-rich sites has been suggested to improve detection [91], although jugular venipuncture remains the most common choice in field studies due to practical considerations. Detection of *Leishmania* spp. is inherently more challenging; optimal molecular diagnosis generally involves tissue samples from lesions (e.g., skin scrapings, needle aspirates, or biopsies) or aspirates from lymph nodes or bone marrow [88,89]. However, such sampling procedures, as well as the collection of peripheral capillary blood, are often impractical under field conditions in equids. For these reasons, jugular vein sampling was adopted for all animals in the present study. While the sampling site is an important parameter for the detection of related parasites, the sampling time-point may also influence the detection of parasites such as *A. phagocytophilum* due to the seasonal pattern of tick activity and pathogen transmission [92,93]. In this study, none of the animals exhibited clinical signs of any diseases, and there were no ticks present on the animals at the time of sampling. Based on the lack of positivity in *A. phagocytophilum*, *Try. evansi* and *Leishmania* spp. in collected equidae samples, we conclude to include alternative sampling sites such as capillary vessels, tissue fluids, biopsies, needle aspirates and skin scrapings in future studies that are aiming to detect these parasites.

## 5. Conclusions

This study determined the prevalence and genetic characteristics of EP in horses from Western Anatolia, Türkiye. The results showed a prevalence of 24.74% (96/388) for *T. equi* and 12.89% (50/388) for *B. caballi*, with all positive horses identified as asymptomatic carriers. These significant prevalence rates highlight the risk posed by carrier animals, which can transmit infection to ticks and may develop clinical disease under immunosuppression, heavy exercise, concurrent illness, or stress. Although no ticks were found on sampled horses, studies targeting tick vectors are needed to assess parasite prevalence. Similarly, more research is required to elucidate the clinical significance of atypical piroplasm infections in equids. Moreover, although other blood parasites such as *Leishmania*, *Trypanosoma*, and *Anaplasma* species were also investigated in this study but not detected, clinicians should consider the potential presence of blood parasites other than *B. caballi* and *T. equi* in the diagnosis and treatment of equine diseases.

## Figures and Tables

**Table 1:** Primer sequences for detection of *Babesia caballi*, *Theileria equi*, *Anaplasma phagocytophilum*, *Trypanosoma* spp. and *Leishmania* spp.

Organisms	Genes	Primer Names	Sequences, 5′-3′	Amplicons Size (Base Pairs)	PCR Conditions	References
* Theileria equi *	18S rRNA	BEC-UF2	TCG AAG ACG A TC AGA TAC CGT CG	435	94 °C 50 s 55 °C 50 s 35 cycle 72 °C 1 m	[39]
		EQUI-R	TGC CTT AAA CTT CCT TGC GAT			
*Babesia caballi* *	* rap-1 *	Bca-F	GAT TAC TTG TCG GCT GTG TCT	222 (nested product)	94 °C 30 s 59 °C 30 s 35 cycle 72 °C 1 m	[40]
		Bca-R	CGC AAG TTC TCA ATG TCA G			
		BcaN-F ^b^	GCT AAG TAC CAA CCG CTG A			
		BcaN-R ^b^	CGC AAG TTC TCA ATG TCA G			
*Anaplasma phagocytophilum* *	16S rRNA	ge3a	CACATGCAAGTCGAACGGATTATTC	546 (nested product)	94 °C 50 s 55 °C 50 s 35 cycle 72 °C 1 m	[41]
		ge10r	TTCCGTTAAGAAGGATCTAATCTCC			
		ge9f ^b^	AACGGATTATTCTTTATAGCTTGCT			
		ge2 ^b^	GGCAGTATTAAAAGCAGCTCCAGG			
*Trypanosoma* spp.	ITS1	Kin1 Kin2	GCG TTC AAA GAT TGG GCA AT CGC CCG AAA GTT CAC C	540 (*Try. evansi*) 300 (*Try. vivax*)	94 °C 1 m 58 °C 1 m 4 cycle 72 °C 1 m 94 °C 1 m 56 °C 1 m 8 cycle 72 °C 1 m 94 °C 1 m 54 °C 1 m 23 cycle 72 °C 1 m	[42]

*Leishmania* spp	LT1	RV1	CTT TTC TGG TCC CGC GGG TAG G	145	94 °C 55 s 55 °C 45 s 35 cycle 72 °C 1 m	[43]
		RV2	CCA CCT GGC CTA TTT TAC ACC A			

* represent species detected by a nPCR approach and ^b^ indicates primers used for the second round of the nPCR. Primer sequences are given in 5′→3′ direction.

**Table 2:** Distribution of *Theileria equi* and *Babesia caballi* infections by age, sex, and breed in equines of Western Aegean Türkiye.

	Factors	Positive Samples (No (%)/(Mean ± Standard Error of The Mean)			
		*T. equi*	*p* Value *	*B. caballi*	*p* Value *
**Age**	≥3 years	54 (25.11%) (1.74 ± 0.43)		19 (8.83%) (1.91 ± 0.28)	
	<3 years	9 (21.43%) (1.78 ± 0.41)		3 (7.14%) (1.92 ± 0.26)	
	Unknown	33 (25.19%) (1.74 ± 0.43)		28 (21.37%) (1.78 ± 0.41)	
			0.871 *^a^		0.002 *
**Sex**	Female	24 (27.27%) (1.72 ± 0.04)		8 (9.09%) (1.90 ± 0.03)	
	Male	35 (25.93%) (1.74 ± 0.03)		10 (7.41%) (1.92 ± 0.02)	
	Unknown	37 (22.42%) (1.77 ± 0.03)		32 (19.39%) (1.80 ± 0.03)	
			0.645 *^b^		0.004 *
**Breed**	Ambler	27 (35.52%) (1.64 ± 0.05)		0 (0%) (2.00 ± 0.00)	
	Arabian	8 (11.43%) (1.88 ± 0.03)		12 (17.14%) (1.82 ± 0.04)	
	English	1 (2.86%) (1.97 ± 0.02)		3 (8.57%) (1.91 ± 0,04)	
	Local breed	12 (60.0%) (1.40 ± 0.11)		4 (20.0%) (1.80 ± 0.09)	
	Haflinger	0 (0%) (2.00 ± 0.00)		0 (0%) (2.00 ± 0.00)	
	Pony	2 (50%) (1.50 ± 0.28)		1 (25%) (1.75 ± 0.25)	
	Donkey/Local	9 (42.86%) (1.57 ± 0.11)		0 (0%) (2.00 ± 0.00)	
	Mule/Local	2 (11.76%) (1.88 ± 0.08)		0 (0%) (2.00 ± 0.00)	
	Unknown	35 (24.65%) (1.75 ± 0.03)		30 (21.13%) (1.78 ± 0.03)	
			0.000 *		0.000 *

* Indicates *p* values considered as statistically significant (<0.05) based on the Kruskal–Wallis test for non-normally distributed data. *^a^ *p* value between age for *T. equi* positive samples *^b^ *p* value between sex for *T. equi* positive samples.

**Table 3:** Demographic distribution of *Theileria equi* and *Babesia caballi* infections.

	Factors	Positive Samples (No (%)/(Mean ± Standard Error of The Mean)			
		*T. equi*	*p* Value *	*B. caballi*	*p* Value *
**Province**	Aydın	58 (32.77%) (1.67 ± 0.03)		8 (4.52%) (1.95 ± 0.01)	
	Denizli	8 (15.09%) (1.84 ± 0.04)		9 (16.98%) (1.83 ± 0.05)	
	İzmir	9 (10.71%) (1.89 ± 0.03)		33 (39.29%) (1.60 ± 0.05)	
	Muğla	21 (28.38%) (1.71 ± 0.05)		0 (0%) (2.00 ± 0.00)	
			0.000 *		0.000 *
	**Overall**	**96 (24.74%)**		**50 (12.89%)**	0.896 *^c^

* Indicates *p* values considered as statistically significant (<0.05) based on the Kruskal–Wallis test for non-normally distributed data. *^c^ *p* value between *T. equi* and *B. caballi* positive samples.

## Data Availability

Dataset available on request from the authors.

## Supplementary Materials

The following supporting information can be downloaded at: https://www.mdpi.com/article/10.3390/vetsci12090826/s1, Table S1. Estimated and actual sample sizes for each sampling region.

## Abbreviations

The following abbreviations are used in this manuscript:

EP	Equine piroplasmosis
EGA	Equine granulocytic anaplasmosis
ECL	Equine cutaneous leishmaniasis
PCR	Polymerase Chain Reaction
n-PCR	Nested Polymerase Chain Reaction
DNA	Deoxyribonucleic acid
