# Supplementary material for: Molecular Detection and Prevalence of Equine Piroplasmosis and Other Blood Parasites in Equids of Western Aegean Türkiye
Source: Vet Sci. 2025 Aug 27;12(9):826. doi: 10.3390/vetsci12090826 (PMC12474372; doi:10.3390/vetsci12090826)
Supplement: Supplementary file 1 [file vetsci-12-00826-s001.zip › vetsci-3793919-supplementary.pdf]

Table S1. Estimated and actual sample sizes for each sampling region.

**Sampling region**	**Confidence level used**	**Confidence limits (*d*)**	**Hypothesized % frequency of outcome factor in the population (*p*)**	**Design effect (for cluster surveys-*DEFF*)**	**[a]Population size (*N*) / [b]Calculated sample size (*n*)**	**Number of animals sampled (*n*)**
İzmir	95%	10%	50%	1	254 / 70	84
Aydın	95%	10%	50%	1	488 / 81	177
Denizli	90%	10%	50%	1	59 / 37	53
Muğla	95%	10%	50%	1	392 / 78	74

([a]); The current number of equids in each province (N) was obtained from the Ministry of Agriculture and Forestry of the Republic of Türkiye.

([b]); Relative sample sizes were calculated using OpenEpi software (Dean A, Sullivan K, Soe M. *OpenEpi: Open-Source Epidemiologic Statistics for Public Health*. 2013. Available from: https://www.openepi.com/Menu/OE_Menu.htm).
